# The BTBR Mouse Model of Autism Spectrum Disorders Has Learning and Attentional Impairments and Alterations in Acetylcholine and Kynurenic Acid in Prefrontal Cortex

**DOI:** 10.1371/journal.pone.0062189

**Published:** 2013-04-24

**Authors:** Stephanie M. McTighe, Sarah J. Neal, Qian Lin, Zoë A. Hughes, Daniel G. Smith

**Affiliations:** Neuroscience Research Unit, Pfizer Global Research and Development, Cambridge, Massachusetts, United States of America; Roma Tre University, Italy

## Abstract

Autism is a complex spectrum of disorders characterized by core behavioral deficits in social interaction, communication, repetitive stereotyped behaviors and restricted interests. Autism frequently presents with additional cognitive symptoms, including attentional deficits and intellectual disability. Preclinical models are important tools for studying the behavioral domains and biological underpinnings of autism, and potential treatment targets. The inbred BTBR T+tf/J (BTBR) mouse strain has been used as an animal model of core behavioral deficits in autism. BTBR mice exhibit repetitive behaviors and deficits in sociability and communication, but other aspects of their cognitive phenotype, including attentional performance, are not well characterized. We examined the attentional abilities of BTBR mice in the 5-choice serial reaction time task (5-CSRTT) using an automated touchscreen testing apparatus. The 5-CSRTT is an analogue of the human continuous performance task of attention, and so both the task and apparatus have translational relevance to human touchscreen cognitive testing. We also measured basal extracellular levels of a panel of neurotransmitters within the medial prefrontal cortex, a brain region critically important for performing the 5-CSRTT. We found that BTBR mice have increased impulsivity, defined as an inability to withhold responding, and decreased motivation, as compared to C57Bl/6J mice. Both of these features characterize attentional deficit disorders in humans. BTBR mice also display decreased accuracy in detecting short stimuli, lower basal levels of extracellular acetylcholine and higher levels of kynurenic acid within the prefrontal cortex. Intact cholinergic transmission in prefrontal cortex is required for accurate performance of the 5-CSRTT, consequently this cholinergic deficit may underlie less accurate performance in BTBR mice. Based on our findings that BTBR mice have attentional impairments and alterations in a key neural substrate of attention, we propose that they may be valuable for studying mechanisms for treatment of cognitive dysfunction in individuals with attention deficits and autism.

## Introduction

Autism spectrum disorders (ASD) are a continuum of neurodevelopmental disorders characterized by core symptom domains of social impairment, communication abnormalities and restricted and repetitive patterns of behavior [1]. Currently, it is estimated that 1 in 88 children in the US are diagnosed with ASD by the age of eight [2]. The impact of ASD on families, individuals and society is profound; many ASD patients never work and need life-long educational and social support. It is estimated that the cost of treatment and care for individuals with ASD in the US reaches $137 billion annually [3], making it a significant public health problem. A greater understanding of the behavioral characteristics, biological underpinnings and potential treatments is therefore a high priority in the autism research and medical communities.

In addition to the core symptom domains, patients with ASD may exhibit co-morbid features such as intellectual disability (ID) and deficits in attention. It is estimated that between 25–75% of ASD patients exhibit ID [4], [5], and although DSM-IV precludes concurrent diagnoses of ASD and attention deficit hyperactivity disorder (ADHD), current estimates of co-morbidity range from 41–78% [6]. Many more individuals with ASD may have attentional deficits that are distinct from those characterizing ADHD. Some risk factors associated with ASD, including multiple genetic mutations, are also associated with ID and ADHD [7]–[9], implying overlap in biological etiology. Perhaps more important is that co-morbid ID [10] and ADHD [11]–[13] can negatively impact patient outcomes; indeed, attentional function in patients with fragile X syndrome has been shown to be predictive of their intellectual development [14]. Overall, consideration of common co-morbidities is important for improving the quality of life for patients with neurodevelopmental disorders.

Preclinical research models are essential for investigating behavioral phenotypes and underlying pathophysiology, and for developing new therapies. The BTBR mouse is an inbred mouse strain with face validity as a preclinical model of the core autism symptom domains, namely decreased social preference [15]–[20], abnormalities in ultrasonic vocalization [21]–[23] and repetitive grooming behavior [20], [24]. Some groups have assessed learning and memory in BTBR mice using reversal learning as a model of restricted and repetitive interests [15], [25], and a recent study found deficits in executive control [26]. However, no study has thus far examined attentional function in any mouse model of autism, nor the underlying neural mechanisms. We aimed to characterize attentional performance and prefrontal cortex neurotransmission in BTBR mice, and determine their utility as an animal model of attentional and learning dysfunction in ASD. We chose to use the 5 choice serial reaction time task (5-CSRTT), as this task is a translational analogue of the human continuous performance test [27]. The 5-CSRTT has a rich history, having been used for nearly thirty years to assess brain regions and neurotransmitter systems underlying attentional processes in rats and mice (for review see [28]). This history is a significant advantage, because the wealth of existing data can be used to guide the interpretation of data gathered using disease models.

We implemented the 5-CSRTT in an automated touchscreen apparatus [29]–[31], a relatively new technology that confers the advantages of increased control of stimulus presentation, and translational relevance to human computer touchscreen testing [32]. The automated touchscreen paradigm retains the automation and control of reward delivery of the original nine-hole box implementation. The touchscreen has been used to assess attention in mouse models [33], [34], but this is the first report of attentional ability in any mouse model of autism behavioral symptoms. To explore key neural substrates of attentional performance, we used *in vivo* microdialysis to measure basal extracellular neurotransmitter levels in medial prefrontal cortex (mPFC), an important brain region for many aspects of 5-CSRTT performance [28].

## Materials and Methods

### Ethics Statement

All procedures related to animal care and treatment were conducted with the approval of the Institutional Animal Care and Use Committee at Pfizer Global Research and Development, Groton CT; and according to the guidelines National Research Council Institute for Laboratory Animal Research Guide for the Care and Use of Laboratory Animals; and the US Department of Agriculture Animal Welfare Act and Animal Welfare Regulations. This study was conducted in accordance with the Guide for the Care and Use of Laboratory Animals as adopted and promulgated by the NIH (Pub. 85–23, revised 1996) and was approved by Institutional Animal Care and Welfare Committee at Pfizer.

### Animals

Male BTBR T+ tf/J (BTBR) and C57Bl/6J (C57) mice from Jackson Laboratories (Bar Harbor, ME, USA), aged 8 weeks on arrival and housed four per cage (individually ventilated cages, measuring 19 cm by 30 cm by 13 cm; Allentown Inc., NJ, USA) were used in all experiments. Mice were kept on a 12 hour/12 hour light cycle (lights off at 6 pm) in a temperature and humidity controlled vivarium. Mice were allowed to acclimate to the vivarium for at least 1 week before testing, and were identified by tail tattoo. Prior to the onset of touchscreen testing, mice were food deprived to 85% of free-feeding weight. Mice were weighed daily and fed accordingly to maintain the 85% target body weight, in line with Animal Use Protocols in place. Water was available *ad libitum* throughout. For each measurement, 12 BTBR and 12 C57 mice were used, except where stated.

### Apparatus

**Figure 1:**
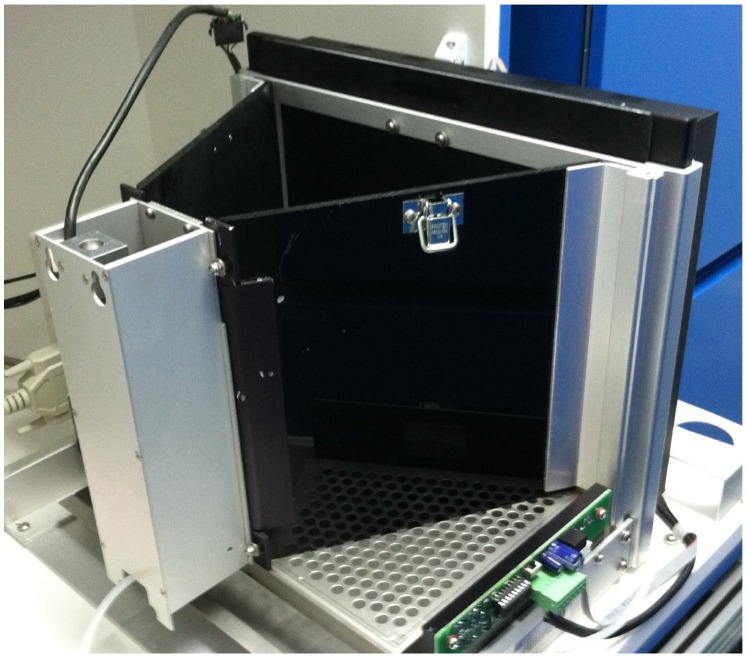
The touchscreen apparatus. The touchscreen apparatus as used in this experiment consisted of a touch sensitive screen and operant chamber mounted within a sound attenuating box and equipped with food reward delivery, a house light, a magazine light and tone generator (Campden Instruments, Ltd., Leicester, UK).

Testing was conducted in a touchscreen-operant training apparatus built into a sound and light attenuating box (Campden Instruments, Ltd., Leicester, UK; figure 1). The apparatus consisted of an acrylic trapezoid shaped enclosure measuring 25.5 cm wide at the widest point, 7.5 cm wide at the narrowest point, 17.5 cm long and 20 cm in height. Two infra-red beams were mounted inside the enclosure to detect the animal’s locomotor activity (a rear beam near the food magazine, and a front beam close to the touchscreen). A clear Perspex lid covered the open top of the enclosure to prevent animals from escaping while still enabling the use of a ceiling mounted camera and a 3W houselight attached to the inside ceiling of the sound attenuating box. A tone generator was mounted 13.5cm above each touchscreen chamber.

Stimuli were displayed on a touch sensitive screen measuring 24.5 cm wide by 18.5 cm high mounted at the widest part of the enclosure. An acrylic mask installed over the touchscreen had 5 cut-outs measuring 4 by 4 cm that defined the response windows. On the opposite (narrow) wall was an opening containing a food magazine, an infra-red beam to detect head-entries, and a light to illuminate the food magazine. Food reward (2% strawberry milk; The Hershey Company, PA, USA) was dispensed to the food hopper from a glass container using a peristaltic pump attached to 0.5 mm bore tubing, providing a dispensing rate of approximately 0.025 ml/s (see Campden Instruments Ltd.: http://www.campden-inst.com/).

The inputs and outputs of the operant box were controlled by a computer running WhiskerServer software [35] (Cambridge University Technical Services Ltd., Cambridge, UK) and ABET II software (Campden Instruments, Ltd., Leicester, UK). Data were collected and analyzed using ABET software, and statistics were performed using GraphPad Prism (GraphPad Software, Inc., La Jolla, CA, USA).

### Grooming Analysis

BTBR mice have been seen in multiple studies to show a higher level of grooming than C57 mice [24], [36], [37]. To ensure that this behavior was not disrupting touchscreen performance, all mice in the study were assessed for grooming behavior. We measured grooming in a clean novel cage environment and in the touchscreen after training on the 5-CSRTT.

Prior to the onset of food restriction or touchscreen training, grooming was assessed in a 20 minute session. Consistent with the literature [19], [20], [24], grooming scoring took place in a clean mouse shoebox cage (measuring 19 cm by 30 cm by 13 cm; Allentown Inc., NJ, USA). Mice were placed individually into a cage and allowed to habituate for 10 minutes. After this time, grooming was scored by a trained experimenter for 10 minutes, using a stopwatch.

After mice had received training on the touchscreen task, grooming was also assessed in the touchscreen box. Each mouse was given one 20 minute session in the touchscreen. The parameters used for this session were 4 second stimulus duration and 5 second ITI, with a maximum of 50 trials (with the aim that animals would be performing the task throughout the grooming assessment). Again the first 10 minutes were treated as habituation, and the second 10 minutes were scored by a trained experimenter, using a stopwatch, from videos recorded using the camera inside the touchscreen box. Time spent grooming was recorded in seconds out of the total 10 minute period.

### Initial Touchscreen Pre-Training

Prior to training on the 5CSRTT, animals (n = 12 BTBR and n = 12 C57 mice) were initially trained to touch the screen. Similar procedures have been used in previous implementations of the 5CSRTT in the mouse touchscreen [33], [34]. Animals received 3–7 habituation sessions until they reached a group criterion of an average of 90 rewards in 60 minutes. Animals then received one session of Pavlovian training. The purpose of this session was to establish an association between the tone and food reward to allow the tone to signal a correct screen touch. In this session, a stimulus (white rectangle) was randomly displayed in one of the five response windows for 30 seconds. After this time, the stimulus would disappear accompanied by food delivery, the tone and illumination of the magazine light. The inter-trial interval before the stimulus would reappear in a new location was 30 seconds. Mice were not required to touch the screen to receive a reward. The houselight remained on throughout. Mice received 50 rewards in a time limit of 60 minutes.

After this, mice were trained to touch the screen in two stages of increasing difficulty. In the first stage, a stimulus was randomly displayed in one of the five response locations. If the mouse touched the stimulus, the food reward was delivered along with the tone and magazine light. There was no response if the mouse responded to any other (non-lit) response window. After reaching criterion on this stage (50 touches in 60 minutes), mice moved onto a punished version of the task. In this stage, touching an unlit square would trigger a timeout period of five seconds, where the houselight would be extinguished and no responses were possible. After this, the same response location would be lit repeatedly until the animal made a correct response (a “correction trial” procedure). This was to help prevent formation of any side biases by forcing the animal to receive rewards by responding to each location. Animals were required to complete 50 trials in 60 minutes, with an accuracy of 80% or greater for two consecutive days to successfully complete pre-training.

### 5 Choice Serial Reaction Time Training

After pre-training, mice moved onto 5 choice training (5-CSRTT training). The 5-CSRTT has been extensively described previously in both rats and mice [38]–[40], and the touchscreen procedure was run in a similar way to the procedure in the 5 or 9 hole operant box [27], [38] and previous touchscreen testing [33], [34].

Each trial began with illumination of the food magazine light. When the mouse made a nose poke in the food magazine, this initiated an inter-trial interval (ITI) period of 5 seconds. During this time no stimuli were displayed on the screen; if the animal touched the blank screen, the trial was recorded as a premature response. After the ITI period, a stimulus appeared in one of the response windows for a set stimulus duration (this varied from 32 to 0.8 seconds in this study). Following the stimulus duration was a limited hold period when no stimuli were displayed but the screen was still active and responses could be recorded. Stimulus durations above 5 seconds had no limited hold, but for durations shorter than 5 seconds, the limited hold period was adjusted so that the animal had five seconds to respond to the stimulus (e.g., for a 4 second stimulus duration, the limited hold was 1 second; for a 0.8 second stimulus duration, the limited hold was 4.2 seconds). This was to allow the animal time to physically respond to the screen after detecting the stimulus. Animals could make two types of responses during the stimulus and limited hold period – correct (touching the lit square) or incorrect (touching any other square). Accuracy was computed by correct responses divided by the total of correct and incorrect responses. If the animal did not make a response over the combined period of the stimulus duration and limited hold, the trial was counted as an omission. Each trial could therefore be classed as premature, correct, incorrect, or an omission. Independently of this, animals could also make perseverative responses, which are screen-touches after a correct or incorrect response. These were recorded separately from the total trials, as animals could make multiple perseverative responses per trial.

Animals started on a stimulus duration of 32 seconds and ITI of 5 seconds. With a goal to baseline mice at a stimulus duration of 0.8 seconds, the stimulus duration was sequentially reduced from 32, 16, 8, 4, 2, 1.8, 1.6, 1.4, 1.2, 1 and 0.8 seconds. Animals had to reach a criterion of completing 50 trials at 80% accuracy and less than 20% omissions in 60 minutes to pass from one stage to the next. The number of trials required to complete was later reduced to 30 as BTBR mice were not completing 50 trials.

### 5 Choice Serial Reaction Time Testing

Animals were given probe sessions to test for aspects of the phenotype.

#### 1. Premature probe sessions

Animals were given a session with a long ITI (10 seconds) to promote premature responding. This manipulation has been used in the past to exacerbate and highlight differences in premature responding in impulsive animals [41]. BTBR and C57 mice were given a baseline session with stimulus duration of 8 seconds and ITI of 5 seconds, then a probe session with stimulus duration 8 seconds and ITI 10 seconds, followed by two more baseline sessions and a second probe session. Mice received a maximum of 30 trials or 50 minutes. Results from the two probe sessions were averaged and compared to the average of the three baseline sessions.

#### 2. Accuracy probe

Animals were given sessions with reduced stimulus durations to test for deficits in attentional accuracy. Animals were baselined at 8 seconds stimulus duration with a 5 second ITI. They were then given four probe sessions at 4, 2, 0.8 and 0.4 seconds stimulus duration. Each probe session was followed by a baseline session of 8 seconds stimulus duration. Animals received a maximum of 30 trials or 45 minutes. The order of probe sessions was counterbalanced in a Latin square design to counteract any order or learning effects.

### *In vivo* Microdialysis

Male BTBR T+ tf/J and C57Bl/6J mice aged 8 weeks (Jackson Laboratories, Bar Harbor, ME) were anesthetized under 2.5% isoflurane to allow stereotaxic implantation of a microdialysis guide cannula targeting the medial prefrontal cortex (mPFC; coordinates relative to bregma: anterior-posterior +2.0 mm; medial-lateral −0.3 mm; dorsal-ventral −0.5 mm [42]). A 2 mm probe (BASi part# MD-2212, West Lafayette, IN, USA) was inserted through the guide cannula and continuously perfused with ACSF (147 mM NaCl, 1.3 mM CaCl_2_, 2.7 mM KCl, 1.0 mM MgCl_2_; 0.5 µl/min). The day after surgery, basal microdialysates were collected for a 60 minute period, and aliquots were taken from this for the analysis of each neurotransmitter. These aliquots were then frozen for subsequent measurement of acetylcholine, histamine, glutamate, kynurenic acid, norepinephrine, dopamine and serotonin. Samples were analyzed by LC-MS/MS, using previously published methods [43]–[46].

### Data Analysis

Data were collected by the automated touchscreen system. Initial analysis was performed using the in-built analysis tool and report editor in ABET II (Campden Instruments, Ltd., Leicester, UK). Statistical analysis was performed using GraphPad Prism (GraphPad Software, Inc., La Jolla, CA, USA). Data from the touchscreen measures were analyzed using repeated measures twoway ANOVA, and t tests, as appropriate. Welch’s correction was used where samples were found to have unequal variance. T-tests were used to compare levels of each neurotransmitter between BTBR and C57 mice.

## Results

### Grooming Analysis

**Figure 2:**
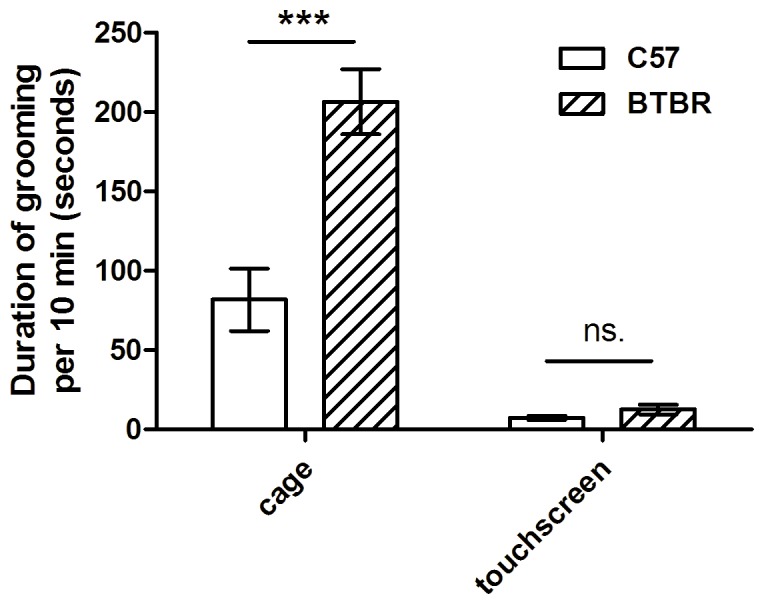
BTBR mice show increased grooming in a cage, but not in the touchscreen. BTBR mice (n = 11) spend significantly more time grooming in the home cage test, but grooming is greatly reduced and not significantly different from C57 mice (n = 10) when both strains are assessed in the touchscreen apparatus. Grooming is shown as the total number of seconds spent grooming in a ten minute test period.

BTBR (n = 11) and C57 (n = 10) mice were assessed for grooming before training and after the last probe trial test in the 5-CSRTT. Due to malocclusion (n = 2) and being underweight (n = 1), 2 C57 and 1 BTBR mice were euthanized prior to the second grooming measurement, leaving n = 11 BTBR and n = 10 C57 mice included in the final analysis. Only mice that had completed both grooming assessments were included in the analysis. Repeated measures ANOVA using location as a within-subjects factor and strain as a between-subjects factor revealed a significant main effect of location (F_(1,19)_ = 82.96, p<0.0001; figure 2), a significant main effect of strain (F_(1,19)_ = 21.40, p<0.0005), and a significant interaction (F_(1,19)_ = 16.39, p<0.001). Post-hoc Bonferroni corrected t tests showed that BTBR mice spent significantly more time grooming than C57 mice in a novel home-cage (t_18_ = 6.12, p<0.001), but that there was no difference when the mice were tested within the touchscreen apparatus t_18_ = 0.26, p>0.05). This suggests that BTBR mice are not grooming excessively when engaged in performing the touchscreen task, and that deficits in attention are likely to be due to cognitive deficits, rather than grooming precluding animals’ performance.

### Initial Touchscreen Pre-Training

#### Habituation to the touchscreen apparatus

Age-matched, free-feeding BTBR mice are generally larger than C57 mice, so 85% weights for age-matched mice were significantly greater for BTBR compared to C57 mice (BTBR: 26.9±0.6 g; C57∶21.3±0.4; shown as mean ± standard error of the mean (SEM) throughout; t_22_ = 8.19; p<0.0001). C57 mice also required less rodent chow to maintain these weights, (ranging from 1.5–2 g/day) as compared to BTBR mice (ranging from 2.5–3 g/day).

**Figure 3:**
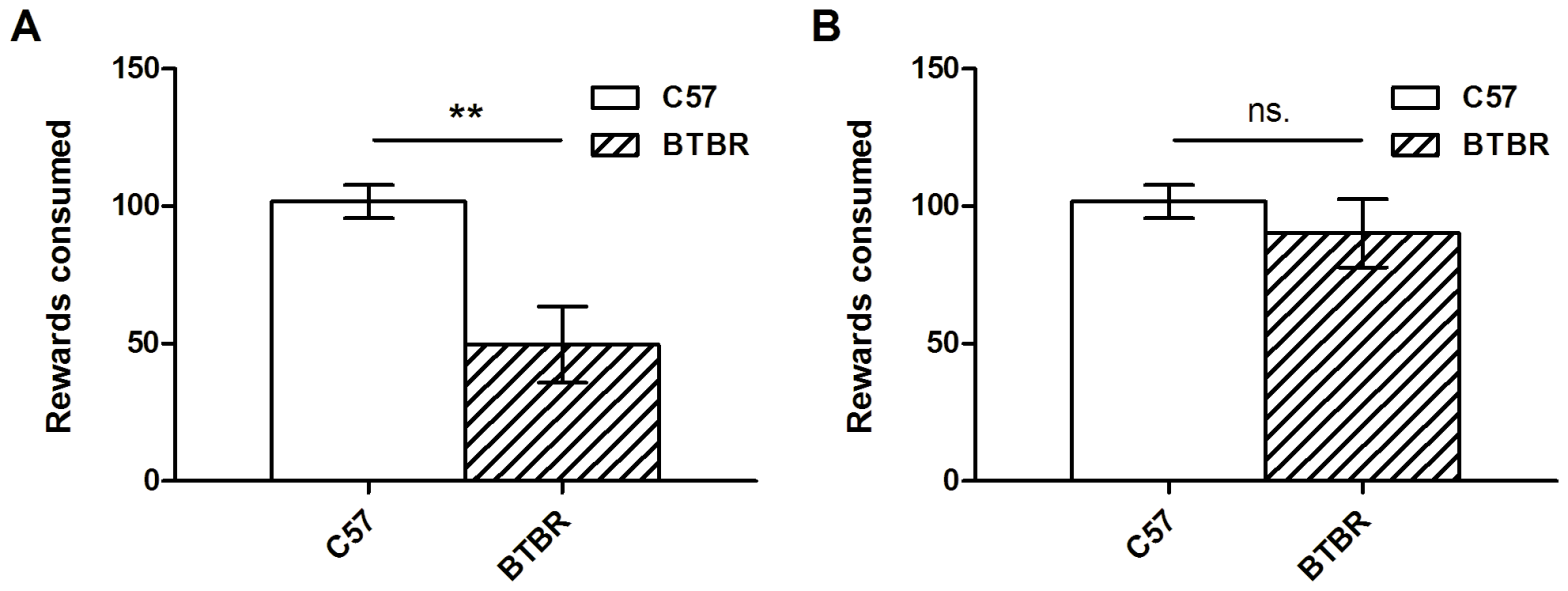
BTBR mice show slower habituation. (A) BTBR (n = 12) mice consume significantly fewer rewards than C57 mice (n = 12) after three days of habituation to the touchscreen. Data are shown for day 3 of habituation. (B) After additional days of habituation, both BTBR and C57 mice are consuming the same number of rewards on the last day of habituation. Data are shown for the last day of total habituation (day 3 for C57 mice, and day 3–8 for BTBR mice depending on individual performance).

BTBR mice obtained and consumed significantly fewer rewards than C57 mice (BTBR: 49.7±14.5; C57∶101.7±6.3; t_15_ = 3.44; p<0.01; figure 3A) during the third of three habituation sessions of 60 minutes. This may be a confound, as if BTBR mice are poorly habituated to food reward, then they may be poorly motivated to perform the task, which will adversely affect learning. In order to equalize responding in BTBR and C57 mice, the habituation criterion was changed to a group criterion of an average of over 90 rewards in 60 minutes. C57 mice and BTBR mice achieved criterion in 3 or 4.5±0.6 days, respectively, and in the last day of this extended habituation, reward consumption was not significantly different across strains (t_15_ = 0.83; p>0.1; figure 3B).

#### Touchscreen training

**Figure 4:**
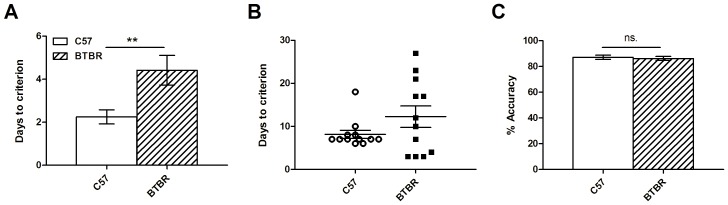
BTBR mice show slower initial learning. BTBR mice (n = 12) took a significantly greater number of days to learn the initial screen-touch, as compared to C57 mice (n = 12; A). There was no significant difference in the number of days taken to learn the “punished” stage of the initial training (B) however the variability exhibited by the BTBR mice is very large compared to C57 mice. Finally, after pre-training was complete, both BTBR and C57 mice showed comparable accuracy performance in the last trial (C) indicating that their performance levels were equal before commencing training on the 5-CSRTT.

All mice successfully ate 50 rewards in 60 minutes in the single Pavlovian conditioning session. In initial touch training, C57 mice were significantly faster than BTBR mice to reach criterion of 50 touches in 60 minutes (t_22_ = 2.83; p<0.01; C57∶2.3±0.3 sessions; BTBR: 4.4±0.7 sessions; figure 4A), and may reflect a subtle deficit in instrumental conditioning in BTBR mice.

In the final (“punished”) stage of pre-training, C57 mice reached criterion in 8.2±1.0 days, and BTBR mice in 12.3±2.6 days. This took 380.8±39.9 trials and 107.4±14.3 errors for C57 mice, and 453.8±82.8 trials, and 126.3±27.6 errors for BTBR mice. There were no significant differences between any of these measures (days: t_22_ = 1.54; p>0.1; trials: t_22_ = 0.83; p>0.1; errors: t_22_ = 0.64; p>0.1; days to criterion are shown in figure 4B).

At the end of pre-training, both C57 and BTBR mouse groups had reached equivalent levels of performance, with C57 mice achieving 87.2±1.7% correct and BTBR mice achieving 86.2±1.7% in the final session of pre-training where they reached criterion (t_22_ = 0.43; p>0.1; figure 4C).

### 5 Choice Serial Reaction Time Training

**Figure 5:**
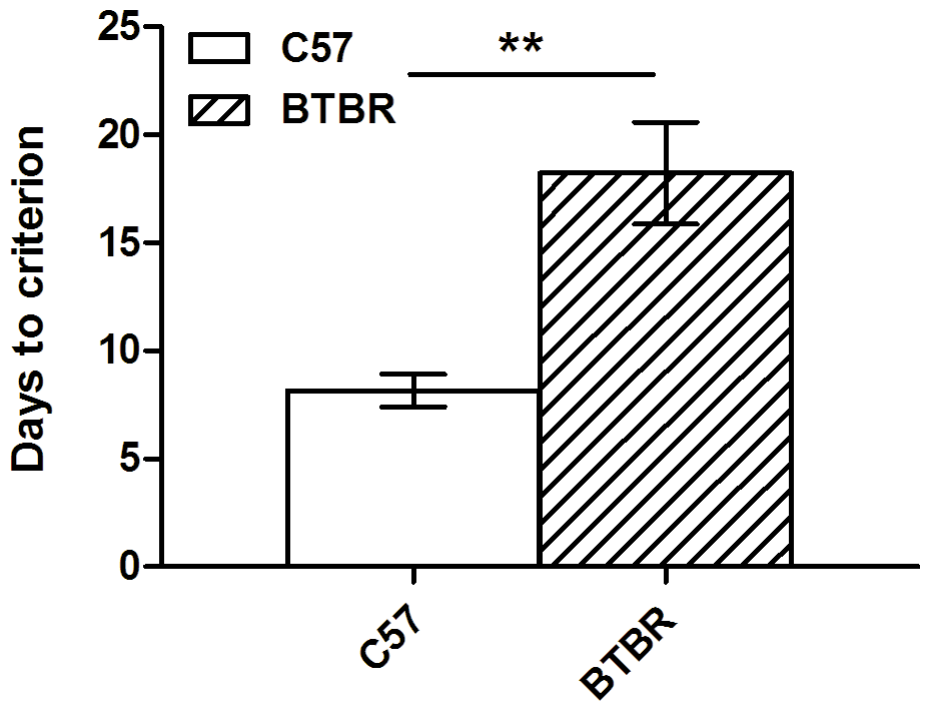
BTBR mice acquire the 5 choice serial reaction time task (5-CSRTT) more slowly than C57 mice. BTBR mice (n = 9) took a significantly greater number of days than C57 mice (n = 12) to reach criterion of 80% accuracy with <20% omissions on the 5-CSRTT at a stimulus duration of 4 seconds.

Three BTBR mice did not reach criterion at 4 seconds stimulus duration. The nine remaining BTBR mice took significantly more days to reach criterion of 80% correct with less than 20% omissions at the 4 second stimulus duration stage (t_9_ = 4.08, p<0.005). BTBR mice took on average 18.22±2.49 sessions, whereas C57 mice took only 8.17±0.80 sessions (figure 5).

These mice reached similar levels of performance after training on the 4 second stimulus duration, with BTBR mice achieving 92.57±1.75% accuracy with 15.11±1.18% omissions, and C57 mice achieving 92.10±1.09% accuracy and 12.83±1.57% omissions. As all animals reached criterion on 8 seconds, this was used as the stimulus duration for baseline sessions during the probes. BTBR mice took a greater number of sessions to reach criterion at 8 second stimulus duration (t_11_ = 3.32, p<0.01), taking on average 10.90±1.78 sessions, whereas C57 mice took 5.08±0.47 sessions.

### 5 Choice Serial Reaction Time Testing

#### 1. Premature probe

**Figure 6:**
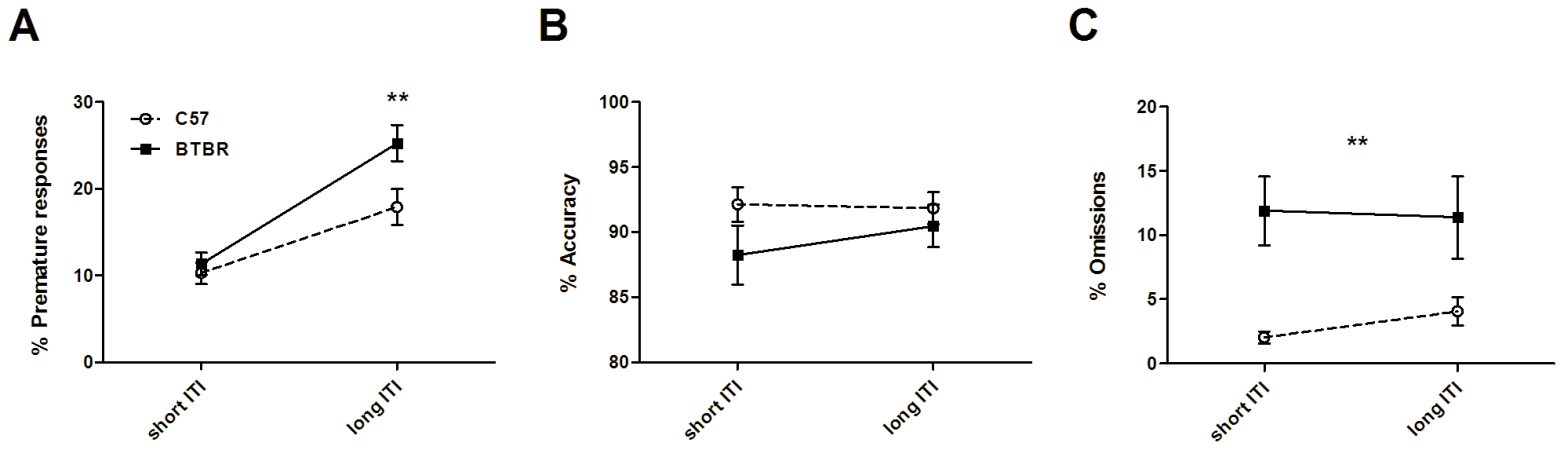
BTBR mice show increased impulsivity. On a long ITI probe session (10 second ITI; 8 second stimulus duration), BTBR mice (n = 12) showed a greater increase in the number of premature responses than C57 mice (n = 12; A). However this manipulation had no effect on accuracy (B) or omissions, although BTBR mice made more omissions in both ITI lengths (C).

The premature probe sessions were conducted with an 8 second stimulus duration and 10 second ITI. Baseline sessions used 8 second stimulus duration and 5 second ITI. Premature responses at 5 second ITI baseline were 11.3±1.3% for BTBR and 10.3±1.3% for C57 mice. At 10 second ITI baseline, BTBR and C57 mice increased their premature responding to 25.3±2.2% and C57 to 17.9±2.1% (figure 6A). Repeated measures ANOVA with within subjects factor of strain and between subjects factor of ITI showed a significant main effect of strain (F_(1,22)_ = 4.52, p<0.05), significant main effect of ITI (F_(1,22)_ = 60.88; p<0.0001) and a significant interaction (F_(1,22)_ = 5.23; p<0.05). Post-hoc Bonferroni corrected t tests showed that BTBR mice made significantly more premature responses than C57 mice in the long ITI condition (t_22_ = 3.05, p<0.01) but were not significantly different at short ITI (t_22_ = 0.44, p>0.1).

There were no significant effects on accuracy (figure 6B; BTBR short ITI: 88.25±2.39%; BTBR long ITI: 90.48±1.70%; C57 short ITI: 92.14±1.40%; C57 long ITI: 91.85±1.29%; main effect of strain; F_(1,22)_ = 1.52; main effect of ITI; F_(1,22)_ = 0.89; interaction; F_(1,22)_ = 1.52; all p>0.1). In contrast, there was a significant main effect of strain on omissions (figure 6C; BTBR short ITI: 11.90±2.79%; BTBR long ITI: 11.39±3.36%; C57 short ITI: 2.04±0.49%; C57 long ITI: 4.06±1.17%; F_(1,22)_ = 8.51; p<0.01) showing that BTBR mice omitted significantly more trials than C57 mice. There was no main effect of ITI or interaction (F_(1,22)_ = 0.72; F_(1,22)_ = 2.00; all p>0.1).

BTBR mice also had significantly longer magazine latencies (short ITI: 1.47±0.06 seconds; long ITI: 1.58±0.08 seconds) than C57 mice (short ITI: 1.15±0.04 seconds; long ITI: 1.15±0.05 seconds; significant main effect of strain; F_(1,22)_ = 27.29; p<0.0001), but there was no main effect of ITI (F_(1,22)_ = 2.16; p>0.1) and no interaction (F_(1,22)_ = 2.24; p>0.1). This pattern of data indicates that BTBR mice show increased impulsivity, as well as decreased motivation.

#### 2. Accuracy probe

**Figure 7:**
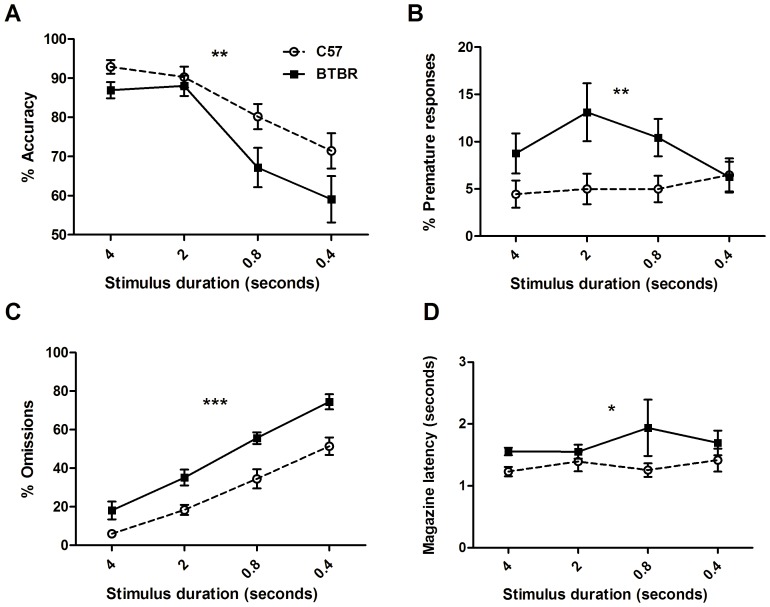
BTBR mice show impaired accuracy, omissions and impulsivity. Performance of BTBR (n = 12) and C57 mice (n = 12) on an accuracy probe session. In these sessions, ITI was held constant at 5 seconds, and mice were given one session at each of 4, 2, 0.8 and 0.4 second stimulus duration. For both strains, accuracy declines as stimulus duration is reduced (A) but BTBR mice are less accurate overall. BTBR mice consistently make more premature responses (B) but there is no effect of stimulus duration. Omissions increase for both strains as stimulus duration is decreased (C), BTBR mice consistently omit more trials than C57 mice. BTBR mice also show consistently longer magazine latencies (D) than C57 mice, again there is no effect of stimulus duration.

Accuracy probes were carried out with 5 second ITI but stimulus durations of 4, 2, 0.8 and 0.4 seconds. As expected, accuracy decreased for both strains when the stimulus duration was shortened (significant main effect of stimulus duration; F_(3,22)_ = 21.65; p<0.0001, figure 7A; for mean and SEM data of all measures, see table S1). There was also a significant main effect of strain, showing that BTBR mice were less accurate (F_(1,66)_ = 9.10; p<0.01), but there was no interaction (F_(3,66)_ = 1.00, p>0.1).

Also as expected, decreasing stimulus duration increased omission rate (demonstrated by a significant main effect of stimulus duration on omissions; F_(3,66)_ = 76.28; p<0.0001, figure 7C). There was a significant main effect of strain on omissions (F_(1,66)_ = 31.79; p<0.0001), showing that BTBR mice omitted more trials than C57 mice, but there was no interaction (F_(3,66)_ = 0.92; p>0.1). BTBR mice showed a greater number of premature responses than C57 mice (significant main effect of strain on premature responses; F_(1,66)_ = 9.29; p<0.01; figure 7B), but there was no main effect of stimulus duration (F_(3,66)_ = 0.84; p>0.1), and no interaction (F_(1,66)_ = 1.67; p>0.1). There was a significant main effect of stimulus duration on perseverative errors (F_(1,66)_ = 5.58; p<0.005), showing that perseverative errors decreased with decreasing stimulus duration. There was no main effect of strain (F_(1,66)_ = 0.90; p>0.1) or interaction (F_(3,66)_ = 0.71; p>0.1).

BTBR mice took significantly longer to retrieve rewards (demonstrated by a significant main effect of strain on magazine latency; F_(1,63)_ = 5.65; p<0.05; figure 7D). There was no main effect of stimulus duration (F_(3,63)_ = 0.42; p>0.1) and no interaction (F_(3,63)_ = 0.66;p>0.1). For all mice, the latency to make a correct response decreased with decreasing stimulus duration (significant main effect of stimulus duration, F_(3,66)_ = 15.11; p<0.0001), but there was no effect of strain (F_(1,66)_ = 0.17; p>0.1) or interaction (F_(3,66)_ = 1.87; p>0.1). BTBR mice responded slower on their incorrect choices than C57 mice (shown by a significant main effect of strain on incorrect response latency; F_(1,42)_ = 11.85; p<0.005), but there was no main effect of stimulus duration (F_(3,42)_ = 0.48; p>0.1) or interaction (F_(3,42)_ = 0.12; p>0.1). Overall, the pattern of data shows that BTBR mice exhibit impaired accuracy, decreased motivation (increased omissions and increased magazine latencies) as well as increased impulsivity.

### *In vivo* Microdialysis

**Figure 8:**
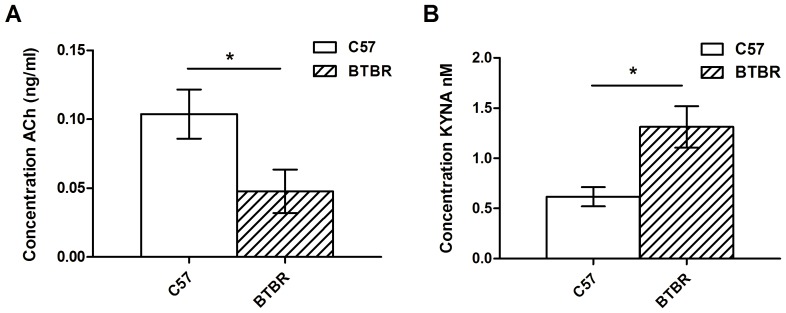
BTBR mice have lower acetylcholine, and higher kynurenic acid levels in mPFC. Basal levels of acetylcholine (A; ACh) and kynurenic acid (B; KYNA) as measured by in vivo microdialysis in mPFC. In BTBR mice (n = 7) levels of acetylcholine were lower and kynurenic acid higher than C57 mice (n = 5) under baseline conditions.

Basal dialysis aliquots were analyzed from 5 C57 and 8 BTBR mice. One BTBR mouse was excluded from the analysis because of aberrant levels of 5-HT in the sample, suggesting blood contamination. Levels of transmitters detected are shown in table 1. The level of acetylcholine was significantly lower in BTBR mice (figure 8A; t_9_ = 2.35, p<0.05), whereas the level of kynurenic acid was significantly higher (figure 8B; t_8_ = 3.05, p<0.05). There were no other significant differences.

**Table 1:** Basal levels of neurotransmitters in medial prefrontal cortex of C57 and BTBR mice.

	C57	BTBR	t	significance	level in BTBR relative to C57
Acetylcholine	0.10±0.02 ng/ml	0.05±0.02 ng/ml	t_9_ = 2.35	*p<0.05	lower
Norepinephrine	0.17±0.08 fM	0.04±0.01 fM	t_4_ = 1.75	ns	
Dopamine	0.44±0.35 fM	0.78±0.70 fM	t_3_ = 0.52	ns	
Histamine	8.20±2.48 ng/ml	5.47±1.35 ng/ml	t_6_ = 1.07	ns	
Glutamate	0.79±0.52 µg/ml	1.05±0.42 µg/ml	t_8_ = 0.42	ns	
Kynurenic Acid	0.62±0.11 nM	1.31±0.22 nM	t_8_ = 3.05	*p<0.05	higher
5-HT	2.30±1.42 fM	1.65±1.04 fM	t_6_ = 0.42	ns	

Basal levels of all neurotransmitters assessed in mPFC in C57 and BTBR mice. Significant differences were seen in acetylcholine and kynurenic acid levels.

## Discussion

It is estimated that between 41–78% of ASD patients have attentional impairments that can adversely affect patient outcomes. Animal models of ASD that exhibit attentional deficits may be useful for understanding attentional dysfunction in ASD, and evaluating new treatments. In the present study, BTBR mice, which are widely used as a model for the core behavioral deficits of ASD, were evaluated for their attentional abilities, as compared to C57 mice. BTBR mice were shown to have impairments in impulse control, motivation and accuracy in detecting short stimuli. They also showed signs resembling neophobia, and a subtle learning deficit during instrumental conditioning training. An assessment of basal neurotransmitter levels in mPFC by *in vivo* microdialysis showed significantly lower levels of acetylcholine and higher levels of kynurenic acid in BTBR mice as compared to C57 mice. These findings highlight alterations in two neurotransmitter systems that have not been previously reported and warrant further investigation in the BTBR mouse.

Importantly, the behavioral deficits in BTBR mice in the 5-CSRTT were not due to performance difficulties caused by their characteristic repetitive grooming behavior. It is important to demonstrate that any impairment in 5-CSRTT performance is due to a cognitive deficit, and not simply due to disruption by other behaviors, including excessive grooming. Under basal conditions, BTBR mice showed robustly increased grooming as compared to C57 mice, consistent with previous studies [19], [20], [24]. In contrast, levels of grooming inside the touchscreen were low, and comparable to C57 mice (figure 2), and therefore unlikely to disrupt task performance. The conditions inside the touchscreen apparatus vary in a number of ways that may explain why increased grooming was not seen in BTBR mice in this environment. Firstly, the mice are on a restricted diet (see Materials and Methods), which provides them with motivation to perform the (food-reward based) task. Secondly, the mice are actively engaged in performing a task, with stimulus presentations and food reward. In standard tests of grooming, mice are not motivated to do any particular action, and their environment is non-stimulating. Food deprivation combined with task demands may have transiently overcome the natural propensity for increased grooming in BTBR mice, enabling us to test them on cognitive tasks.

During initial training to respond using the touchscreen, BTBR mice obtained and consumed significantly fewer rewards (figure 3). The strawberry milk reward and the touchscreen enclosure were novel to the animals at the start of training, and novelty aversion associated with the food or the testing environment may account for the strain-dependent difference. Resistance to change and anxiety associated with novelty are among the features defining the core symptom of restricted interests in individuals with ASD, and the response to novelty in BTBR mice during training in our study may represent an animal analogue of a similar behavioral phenomenon. Using an olfactory task designed to assess restricted, repetitive behaviors in BTBR mice, Moy et al. [47] reported that BTBR mice do not transfer preference to an appetitive olfactory stimulus, even after habituation to the stimulus in the home cage. Those findings and our results are consistent with one another, and suggest that BTBR mice exhibit alterations in how they, as compared to C57 mice, habituate to novel appetitive stimuli.

BTBR mice took longer to learn to respond using the screen in the initial pre-training sessions (figure 4A), suggesting that they may have a subtle instrumental learning deficit. The literature is unclear on whether BTBR mice show a learning deficit; many different tasks with very different demands have been employed, and it is difficult to relate and reconcile the diverse findings. For example, BTBR mice have been shown to be slower to acquire the Morris Water Maze task [15]. However this task places high demand on instrumental conditioning (i.e., associating escape with the platform) and hippocampus-dependent spatial learning (i.e., learning the location of the platform), is aversively motivated, and requires significant locomotion. These demands are very different from those of the current task, which is appetitively motivated, and has a much reduced locomotor component, making these tasks difficult to compare. In a prior touchscreen study, where the initial learning phase more closely resembled the features and demands of initial learning in the present study, Rutz and Rothblat [26] report that BTBR mice do not have a significant initial learning impairment. However, there is an important trend suggesting an impairment in BTBR mice: BTBR mice made nearly double the total number of errors in learning the initial discrimination, when compared to C57 mice (BTBR: 68.2±13.8; C57∶39.3±5.6; p = 0.068). It is notable that in their study and the present study (figure 4B), BTBR mice had substantially greater population variance as compared to C57 mice. Increased population variance in BTBR mouse performance may be a reproducible feature of their phenotype, raising the possibility that evidence for a meaningful learning deficit in some tasks may be masked by variability.

In a probe test using a long ITI designed to challenge impulse control, BTBR mice made significantly more premature responses, as compared to C57 mice (figure 6). This is indicative of increased impulsivity, and was independent of accuracy or omissions, suggesting that the manipulation of increasing ITI selectively increased premature responses in BTBR mice. The impulsive behavior of BTBR mice overlaps with a core symptom domain of ADHD [1], and may implicate similar neuronal substrates. Higher premature responding in the 5-CSRTT is associated with dysfunction of the nucleus accumbens core and infra-limbic prefrontal cortex (see [48] for review), and may implicate circuits centered around these structures in the abnormal impulsivity of BTBR mice. Onaivi and colleagues [49] showed differential release of dopamine in the striatum and frontal cortex, and differential release of serotonin in the striatum of BTBR mice versus C57 mice, after drug administration. However, the potential consequences of this dysregulation on impulsive behavior are not clear. Striatal and frontal systems therefore remain an area for future investigation in the BTBR mouse.

In probe tests designed to assess response accuracy, BTBR mice had worse accuracy performance where decreasing stimulus durations were used (figure 7). This finding suggests that BTBR mice have a lower attentional capacity than C57 mice. Throughout the long ITI premature probe trials and varying stimulus duration accuracy probe trials, BTBR mice consistently showed a greater number of omissions (figures 6 and 7). In the accuracy probes, both mouse strains showed increased omissions at shorter stimulus durations – which is to be expected because as the stimuli grow harder to detect, the number of trials omitted increases. However, BTBR mice omitted more trials at every stimulus duration, suggesting that they have a general motivational deficit rather than increased susceptibility to shorter stimulus durations. BTBR mice also took significantly longer to retrieve rewards (i.e., they had longer magazine latencies) as compared to C57 mice, and further suggests a deficit in motivation. BTBR and C57 mice were both constantly held at 85% of free-feeding weight, and therefore were expected to be equated on motivation for food reward. This phenotype may be very important when considering performance on other appetitively-motivated learning tasks. If there is a baseline deficit in motivation, then this may be a confound when trying to assess the learning abilities of BTBR. Whether this will generalize to aversive motivation, or to other modalities such as social motivation, may be a worthy area for future study. Motivational deficits are a common feature in ADHD, where it can manifest as inattentiveness [50]. It may be possible to capitalize on this feature of the BTBR mouse to model decreased motivation in addition to impulsivity.

BTBR mice were found to have lower extracellular levels of acetylcholine and higher levels of kynurenic acid in mPFC as compared to C57 mice (figure 8). The observation of reduced ACh in mPFC is consistent with the importance of this neurotransmitter in attention and may shed light on the potential mechanism of the impairment of BTBR mice in this task. In rats, excitotoxic lesions of mPFC produce large and long-lasting deficits on accuracy in the 5-CSRTT [51], implicating this structure in accurate performance. The main source of cholinergic inputs to the mPFC is the nucleus basalis magnocellularis in the basal forebrain. Lesions of the nucleus basalis using excitotoxins [52], [53] or 192 IgG saporin, which targets cholinergic neurons [54] both produce accuracy deficits, suggesting that cholinergic function in mPFC is important for accurate performance. Specific blockade of muscarinic cholinergic receptors by infusing the antagonist scopolamine directly into mPFC produces increased omissions [55], [56] and decreased accuracy [56]. It has also previously been shown using both microdialysis and biosensor technology that acetylcholine is released in mPFC during attentional performance [57], [58]. It is possible that deficits in cholinergic transmission in mPFC may account for both decreased accuracy and increased omissions in the BTBR mouse. However, a causal link has not yet been established in this mouse model, and the exact functional significance of lower acetylcholine levels in BTBR mice will require further study. Experiments utilizing optogenetic approaches, intracranial pharmacology manipulating cholinergic transmission, or *in vivo* microdialysis in the behaving animal undergoing attentional tasks will be important next steps to fully determine whether the cholinergic deficit is responsible for any part of the attentional disruption.

BTBR mice also had higher levels of kynurenic acid within the PFC compared to C57 mice (figure 8B). Increased kynurenic acid levels have been reported in the cerebro-spinal fluid of individuals with schizophrenia [59]. Based on this finding, and the ability for kynurenic acid to act as an antagonist at the glycine site of the NMDA receptor, a kynurenic acid hypothesis of schizophrenia has been proposed [60]. While BTBR mice are used as a model of behaviors characteristic of ASD, they have a spontaneous deletion of the *Disc1* gene [61], one of the major genetic risk factors for schizophrenia [62], and the increased kynurenic acid levels and cognitive deficits found in the present study suggest that they may be relevant as a model of phenotypic deficits associated with schizophrenia. In contrast, BTBR mice are reported to show normal sensory gating as tested by prepulse inhibition of startle (PPI) [63], whereas sensory gating deficits are common in individuals with schizophrenia [64]. However, this result is compared to the C57Bl/6J mouse strain, which despite being a standard strain in most behavioral tests, itself displays poor PPI performance [65]. Further phenotypic characterization of BTBR mice is necessary to conclude on whether they exhibit schizophrenia-relevant behaviors.

Increased kynurenic acid levels are reported to have anticonvulsant-like properties, and may play a role in seizure disorders [66], [67]. This may appear inconsistent with the BTBR mouse as a model of ASD, as individuals with ASD have an increased risk of experiencing seizures. However, it is unlikely that increased basal levels of extracellular KYNA within the prefrontal cortex renders the BTBR mouse inconsistent with an animal model of ASD. ASD’s are highly heterogeneous conditions with complex genetic and biological underpinnings and clinical phenotypes [68]. Clinical studies on the rate of epilepsy in individuals with ASD indicate that it is a minority (approximately 20–40%) of patients that exhibit ASD and epilepsy co-occurrence, and the underlying biological etiologies in these individuals are unknown [69], [70]. Preclinical models of non-syndromic ASD also do not consistently exhibit behavioral or electrophysiological seizure phenotypes [71]. BTBR mice have not been reported to exhibit spontaneous seizures, and their susceptibility to induced seizures has to our knowledge not been tested. It may be that the etiology of the pathology of BTBR mice is more consistent with forms of ASD where seizures are not present. Further work including pharmacological manipulation of the kynurenic acid system will be required to conclude on the functional significance of increased kynurenic acid levels in BTBR mice.

There were no significant differences in the levels of monoamines within the mPFC, but this may be due to the fact that basal concentrations of these neurotransmitters are very low as compared to other brain regions, such as the striatum, and in some cases fell below the limits of detection of our analytical systems.

Overall, BTBR mice are less accurate, show greater premature responses and higher omission rates compared to C57 mice, mirroring the inattention (increased omissions) and impulsivity (increased premature responses) features of ADHD. These deficits in accuracy and omissions are accompanied by decreased acetylcholine in the mPFC, which was revealed by microdialysis in the present study. The deficits in attentional processing in BTBR mice suggest that their phenotype, as a model for ASD, spans beyond the core symptom domains. The touchscreen is being widely adopted by the preclinical research community, and has recently been applied to testing mouse models of ASD [26], [72]. It has advantages of throughput and translatability, and broader use will ease comparisons between tasks and investigators. This can only serve to better understand the complex cognitive changes associated with autism animal models, and allow further exploration of common co-morbid symptom domains, including attention and executive function.

## Supporting Information

Table S1 **Mean and SEM data for the accuracy probe trials.**
(DOCX)
