# Supplementary material for: The BTBR Mouse Model of Autism Spectrum Disorders Has Learning and Attentional Impairments and Alterations in Acetylcholine and Kynurenic Acid in Prefrontal Cortex
Source: PLoS One. 2013 Apr 24;8(4):e62189. doi: 10.1371/journal.pone.0062189 (PMC3634761; doi:10.1371/journal.pone.0062189)
Supplement: Table S1 — Mean and SEM data for the accuracy probe trials. (DOCX) [file pone.0062189.s001.docx]

Table S1

| Measure | Stimulus duration (s) | C57 (mean ± SEM) | BTBR (mean ± SEM) |
| --- | --- | --- | --- |
| Accuracy | 4 | 92.91 ± 1.83 | 86.93 ± 2.18 |
|  | 2 | 90.27 ± 2.77 | 88.04 ± 2.72 |
|  | 0.8 | 80.18 ± 3.36 | 67.15 ± 5.24 |
|  | 0.4 | 71.43 ± 4.70 | 59.08 ± 6.18 |
| Omissions | 4 | 5.93 ± 1.98 | 18.1 ± 4.84 |
|  | 2 | 18.33 ± 2.76 | 35.12 ± 4.28 |
|  | 0.8 | 34.46 ± 5.24 | 55.59 ± 3.17 |
|  | 0.4 | 51.38 ± 4.74 | 74.45 ± 4.07 |
| Premature | 4 | 4.44 ± 1.50 | 8.75 ± 2.22 |
|  | 2 | 5.00 ± 1.69 | 13.11 ± 3.20 |
|  | 0.8 | 5.00 ± 1.45 | 10.43 ± 2.06 |
|  | 0.4 | 6.47 ± 1.83 | 6.25 ± 1.69 |
| Perseverative | 4 | 4.92 ± 1.65 | 5.17 ± 1.25 |
|  | 2 | 5.17 ± 1.66 | 2.75 ± 0.78 |
|  | 0.8 | 2.50 ± 1.03 | 2.50 ± 0.66 |
|  | 0.4 | 1.58 ± 0.44 | 0.75 ± 0.32 |
| Magazine latency | 4 | 1.23 ± 0.08 | 1.56 ± 0.06 |
|  | 2 | 1.39 ± 0.16 | 1.56 ± 0.11 |
|  | 0.8 | 1.26 ± 0.11 | 1.91 ± 0.43 |
|  | 0.4 | 1.42 ± 0.19 | 1.70 ± 0.21 |
| Correct latency | 4 | 1.54 ± 0.12 | 1.74 ± 0.14 |
|  | 2 | 1.36 ± 0.11 | 1.37 ± 0.08 |
|  | 0.8 | 1.22 ± 0.07 | 0.97 ± 0.09 |
|  | 0.4 | 1.11 ± 0.12 | 0.99 ± 0.17 |
| Incorrect latency | 4 | 1.67 ± 0.32 | 2.08 ± 0.33 |
|  | 2 | 1.93 ± 0.40 | 2.37 ± 0.43 |
|  | 0.8 | 1.76 ± 0.28 | 2.24 ± 0.32 |
|  | 0.4 | 1.63 ± 0.26 | 2.05 ± 0.35 |
